# Juvenile recurrent parotitis in a 4-year-old patient: a case report

**DOI:** 10.11604/pamj.2021.40.86.27001

**Published:** 2021-10-11

**Authors:** Nouha Dammak, Latifa Berrezouga, Imen Raadani, Wissal Selmi, Mohamed Ben Khelifa

**Affiliations:** 1 Department of Medicine and Oral Surgery, University Dental Clinic of Monastir, Monastir, Tunisia,; 2 Department of Dental Medicine, Fattouma Bourguiba Teaching Hospital, Monastir, Tunisia

**Keywords:** Juvenile recurrent parotitis, ultrasonography, symptoms, treatment, case report

## Abstract

Juvenile recurrent parotitis (JRP) is a rare disease. It is most commonly occurring between the ages of 3 and 5 years, that classically resolves at adolescence. It is characterized by recurrent non-suppurative parotitis, with several acute inflammatory episodes per year. The parotid´s swelling tends to be unilateral, but it can occur bilaterally, with a more predominant side. The aim of this work was to present a case report that highlights signs and symptoms of this unusual condition and to stress on the value of ultrasonography as an aid to diagnosis.

## Introduction

Juvenile recurrent parotitis (JRP) is a rare recurrent inflammation of the parotid glands, occurring in children. It is the second most common inflammatory gland disease after mumps [1]. The incidence of JRP is not precisely determined. The pathogenesis remains obscure and a multifactorial origin might be involved [2]. The diagnosis is based on the history of the disease, the clinical symptoms, and mainly on ultrasonography (USG) which demonstrates multiple hypoechoic zones with vacuolization within the parenchyma [3,4]. Treatment is not yet standardized, but the use of antibiotics and analgesia in acute episodes aims to alleviate the symptoms and to prevent damage to the gland parenchyma. The aim of this report was to highlight signs and symptoms of this unusual condition and to stress on the value of ultrasonography as an aid to diagnosis.

## Patient and observation

**Patient information:** a 4-year-old male patient was referred by his pediatric doctor for restricted mouth opening. According to his mother, symptoms have been evolving, with no fever, since one day following a soft trauma. She also reported the diagnosis of mumps that was performed two months before with the same presenting symptoms. There was neither family history of recurrent parotid swelling or parotid gland problems, nor history of mouth and eyes dryness, joint pains and skin rashes suggestive of autoimmune disorders.

**Figure 1:**
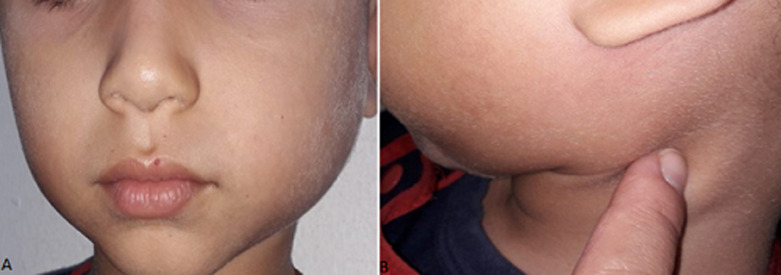
extraoral view: A) swelling of the left parotid region; B) presence of left cervical lymph node

**Figure 2:**
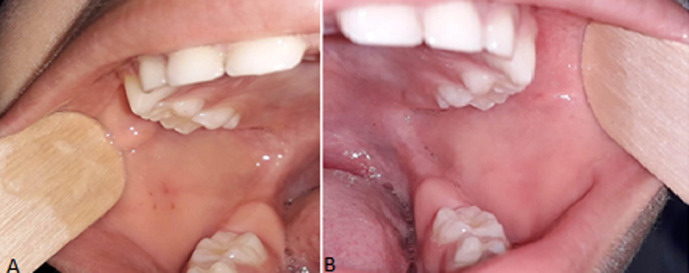
intraoral view showing the absence of caries or other dental anomalies A) right side; B) left side

**Clinical findings:** the extra oral examination (Figure 1) revealed a painful and firm swelling in the left parotid region, and one cervical lymph node enlargement which was oval, mobile and tender on palpation. The intra oral exam (Figure 2) showed an adequate oral hygiene. There was no evidence of caries or other dental anomalies. The mucosa was moist and well lubricated. An erythema around the Stensen´s duct opening was noted and a serious but not purulent discharge was seen on pressing the parotid glands. The diagnosis of viral parotitis was proposed.

**Figure 3:**
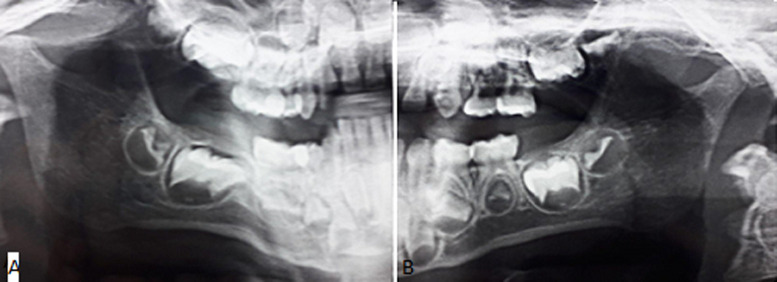
panoramic radiograph showing the absence of dental or alveolar bone anomalies A) right side; B) left side

**Figure 4:**
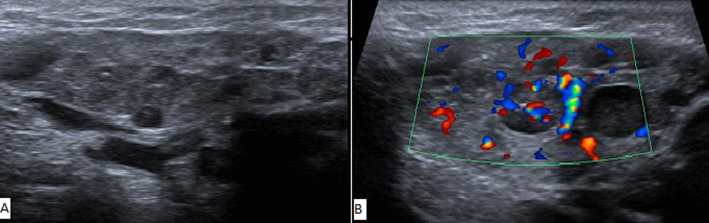
ultrasonography images A) presence of multiple hypoechogenic areas within the parenchyma; B) hypervascularization of the gland parenchyma

**Diagnostic assessment:** a panoramic radiography was performed to search for any dental infectious foci. In fact, no dental or alveolar bone anomalies were displayed (Figure 3). The USG images (Figure 4) showed the presence of multiple hypoechogenic areas and a hypervascularization within the parenchyma gland, confirming the diagnosis of JRP.

**Therapeutic intervention:** the patient was given systemic antibiotics by his pediatric doctor.

**Follow-up and outcomes:** the swelling resolved within 5 days. No other episodes have been seen since 4 months.

## Discussion

The etiopathogenesis of JRP remains unclear and several factors have been suggested for its development including hereditary genetic factors, congenital ductal malformations, bacterial or viral infections, allergy and local manifestation of an autoimmune disease [3,4]. Our case about a 4-year-old boy is in agreement with literature. As this disease usually manifests between 3 and 5 years of age, but earlier and later occurrence have been reported [5]. Leerdam *et al*. [6] described a biphasic age distribution with peaks at 2 to 5 years of age and at 10 years. The disease affects males more than females [7-9], nevertheless, Papadopoulou-Alataki E *et al*. [10] found the same sex distribution ratio in 23 children with JRP. Juvenile recurrent parotitis is characterized by its nonobstructive and nonsuppurative nature [5], recurrent episodes of pain and swelling in the parotid gland, usually associated with fever and malaise [9]. In the present case, no fever was noticed, as reported by Leerdam *et al*. [6] in his study of 53 children with JRP (41.5%), however swelling (100%) and pain (92.5%) were common. As seen in the present case, the parotid gland enlargement is unilateral [5], it occurred in 66% and in 74% according to Papadopoulou-Alataki E *et al*. [10] and Leerdam *et al*. [6], respectively. Rarely, the gland enlargement can be bilateral, with a more predominant side [7,11] and varying degrees of discomfort [8].

The diagnosis of JRP is evoked following the recurrence of the parotid swelling, and is confirmed by USG [12] which is recommended as the gold standard for both diagnosis and follow up. This exam displays multiple small hypoechoic areas corresponding to sialectasis as described in our case. Indeed, USG allows to exclude stones, abscesses and mass lesions. Other investigation tools are used for the diagnosis such as conventional sialography, sialendoscopy, magnetic resonance sialography and magnetic resonance imaging [5]. It´s worth to note that sialography and parotid gland biopsy are no longer indicated because of their invasiveness [12]. Regarding the differential diagnosis, the following diseases involving a parotid swelling should be ruled out like mandibular osteomyelitis, lymphoepithelial cyst, primary Sjogren´s syndrome (SS) , mumps, congenital cystic lesion , sialolithiasis, benign tumours and malignancies (leukaemia, lymphoma) [7]. These tend to present as one-off episodes or persistent recurrent swellings [7]. The previous episodes of swelling, diagnosed as mumps, for our case, could have been an early presentation of JRP, however, this could not be proven as no investigation was carried out. Differentiation between the first episode of JRP and mumps is not an easy task. The notable difference between JRP and mumps, is that while the child develops fever, discomfort, headache, and chills with mumps, the symptoms in JRP are usually more localized in the parotid gland with occasional bouts of fever [9,13]. The most prevalent manifestations of SS are lacrimal and salivary gland dysfunction and parotid swelling. However, these symptoms are not always present at the same time and recurrent parotitis particularly with bilateral involvement appears to be the single most common manifestation in children with SS [7,10]. So the detection of specific antibodies confirm the diagnosis [5].

No preventive therapy against JRP is available [7]. An attention to good oral hygiene, a regimen of massage, fluid intake and use of chewing gum and sialogogues can be helpful in alleviating the symptoms, reducing the recurrence frequency, as can duct probing and dilation [7,9-13]. There is no cure for JRP and the management remains confusing and controversial [5]. The treatment of JRP has changed from invasive to more conservative surgery. Antibiotic use is a subject of controversy as this condition is rarely purulent [11]. In fact, antibiotics and analgesics use in acute episodes aims to prevent additional damage to the glandular parenchyma and to relieve the symptoms [9]. However, there is no evidence that antibiotics affect the duration of episodes [14]. In a systematic review [4] which assesses the role of sialendoscopy in JRP, the authors demonstrated the diagnostic value of sialendoscopy by visualizing strictures, hypovascularization and whitish intraductal debris. They also concluded that sialendoscopy is useful for treatment, by allowing intraductal lavage and, when possible, dilatation of strictures. In a systematic review of treatment studies [1], authors concluded that the most efficient treatment remains unknown and they couldn´t draw definite conclusions. JRP is a self-limiting disease with spontaneous resolution of symptoms after puberty in the majority of cases [5, 9], but may also persist into adulthood [10], as in few reported cases the parotid gland undergo progressive destruction and continue as chronic parotitis [11]. The prognosis of JRP is often favorable. But it does affect the child´s quality of life with poor feeding during attacks and school absenteeism affecting their school activity and social life significantly [11].

## Conclusion

Although JRP is relatively rare, it should be considered in a child suffering from recurrent preauricular swelling. Some signs and symptoms may guide the diagnosis but currently the ultrasonography finds its place to confirm it.
